# A catchment and location-allocation analysis of mammography access in Delaware, US: implications for disparities in geographic access to breast cancer screening

**DOI:** 10.1186/s13058-023-01738-w

**Published:** 2023-11-08

**Authors:** Jessica L. Webster, Neal D. Goldstein, Jennifer P. Rowland, Catherine M. Tuite, Scott D. Siegel

**Affiliations:** 1 Department of Epidemiology and Biostatistics, Drexel University Dornsife School of Public Health, Philadelphia, PA USA; 2 Department of Radiology, Breast Imaging Section, Helen F. Graham Cancer Center & Research Institute, Christiana Care Health System, Newark, DE USA; 3 Cawley Center for Translational Cancer Research, Helen F. Graham Cancer Center & Research Institute, Christiana Care Health System, 4701 Ogletown-Stanton Road, Newark, DE 19713 USA

**Keywords:** Breast cancer, Mammography, Disparity, Catchment, Location-allocation

## Abstract

**Background:**

Despite a 40% reduction in breast cancer mortality over the last 30 years, not all groups have benefited equally from these gains. A consistent link between later stage of diagnosis and disparities in breast cancer mortality has been observed by race, socioeconomic status, and rurality. Therefore, ensuring equitable geographic access to screening mammography represents an important priority for reducing breast cancer disparities. Access to breast cancer screening was evaluated in Delaware, a state that experiences an elevated burden from breast cancer but is otherwise representative of the US in terms of race and urban–rural characteristics. We first conducted a catchment analysis of mammography facilities. Finding evidence of disparities by race and rurality, we next conducted a location-allocation analysis to identify candidate locations for the establishment of new mammography facilities to optimize equitable access.

**Methods:**

A catchment analysis using the ArcGIS Pro Service Area analytic tool characterized the geographic distribution of mammography sites and Breast Imaging Centers of Excellence (BICOEs). Poisson regression analyses identified census tract-level correlates of access. Next, the ArcGIS Pro Location-Allocation analytic tool identified candidate locations for the placement of additional mammography sites in Delaware according to several sets of breast cancer screening guidelines.

**Results:**

The catchment analysis showed that for each standard deviation increase in the number of Black women in a census tract, there were 68% (95% CI 38–85%) fewer mammography units and 89% (95% CI 60–98%) fewer BICOEs. The more rural counties in the state accounted for 41% of the population but only 22% of the BICOEs. The results of the location-allocation analysis depended on which set of screening guidelines were adopted, which included increasing mammography sites in communities with a greater proportion of younger Black women and in rural areas.

**Conclusions:**

The results of this study illustrate how catchment and location-allocation analytic tools can be leveraged to guide the equitable selection of new mammography facility locations as part of a larger strategy to close breast cancer disparities.

**Supplementary Information:**

The online version contains supplementary material available at 10.1186/s13058-023-01738-w.

## Introduction

Breast cancer is the leading cause of cancer incidence and the second leading cause of cancer mortality among US women [1]. Advances in early detection and treatment are largely believed to have contributed to the 40% reduction in breast cancer mortality observed over the last 30 years, [1] but not all groups have benefited equally from these advances. Persistent breast cancer disparities have been observed by race, socioeconomic status (SES), and geographic area. Black women have a 40% higher breast cancer mortality rate relative to White women despite similar incidence rates between the racial groups [1]. This mortality rate grows to 86% higher for younger Black vs. White women [1], owing to the greater risk that Black women have of being diagnosed with advanced-stage breast cancer before age 50 [2]. Other research found 14% lower breast cancer five-year survival rates for low-SES patients relative to their more advantaged peers [3]. Approximately two-thirds of this SES disparity was attributable to conditions at presentation, including later stage at diagnosis. Finally, geographic characteristics including neighborhood measures of disadvantage (e.g., SES, segregation) [4–10] and rurality [11–14] have been associated with later stage at diagnosis and poorer breast cancer survival.

Given the consistent link between later stage at diagnosis and disparities in breast cancer outcomes across multiple population subgroups, ensuring equitable access to screening mammography represents an important goal of breast cancer prevention and early detection. Screening mammography has been a central component of breast cancer programs in the USA over the last 30 + years [15]. A review of the evidence shows that screening mammography can reduce breast cancer mortality by at least 40% when completed on an annual basis beginning at age 40 (vs. women who were not invited to screening) [16–18]. More recent studies have helped to establish that the benefits of screening are independent of treatment advances [19, 20]. Screening mammography decreases mortality by detecting tumors at a smaller size and an earlier stage, when therapy is more effective [21]. There is also preliminary evidence that the benefits of screening mammography extend to the early detection of triple negative breast cancer (TNBC), an aggressive subtype of breast cancer that disproportionately impacts Black women [22].

Decisions about how to allocate mammography resources to ensure equitable geographic access are contingent on which set of screening guidelines are adopted. Multiple US medical organizations have issued screening guidelines that vary along several dimensions, including the recommended age of initiation and screening interval [23]. On one end of the spectrum, the American College of Radiology (ACR) [24] recommends that women of average risk for breast cancer should initiate annual screening mammography at age 40 to maximize life-years gained. The National Comprehensive Cancer Network (NCCN) [25] and the American Society of Breast Surgeons (ASBrS) [26] have issued similar recommendations. On the other end of the spectrum, the US Preventive Services Task Force (USPSTF) published a “B” recommendation (i.e., “moderate to substantial net benefit”) for women of average risk to initiate biennial screening mammography at age 50 [27]. The USPSTF issued a lower level “C” recommendation (i.e., “small net benefit”) for women ages 40–49, citing the need to balance the benefits of screening against the potential harms of overdiagnosis and overtreatment [27]. It should be noted that the USPSTF is currently drafting an update to these recommendations [28]. Other organizations, such as the American Cancer Society (ACS), have issued recommendations that fall somewhere between the ACR and USPSTF guidelines (i.e., annual mammography initiated at age 45 before transitioning to biennial screening beginning at age 55) [29]. Ensuring equitable access to mammography facilities under the ACR relative to the USPSTF guidelines would likely require significantly greater mammography screening capacity given the earlier age of initiation and shorter screening interval, particularly in rural and other disadvantaged areas where access is typically limited.

Beyond the existing screening guidelines, increasing awareness of racial differences in the age distributions of breast cancer incidence and mortality has called for action to advance health equity. While some organizations, such as the ACR and ASBrS, have called for formal lifetime breast cancer risk assessment by the age of 30 for Ashkenazi Jewish and Black/African American women to identify those who would benefit from risk reduction strategies including earlier screening with mammography and/or MRI, some have suggested establishing race-based imaging guidelines [30–32]. Race-based guidelines refer to screening schedules based on a patient’s race. It has been argued that the USPSTF guidelines contribute to racial disparities and should be specifically modified to recommend screening initiation at age 40 for Black women [30]. As noted, the ACR and other organizations do currently recommend screening for all women beginning at age 40, regardless of race. However, under the Affordable Care Act, private insurers and Medicaid are only required to cover preventive services recommended by the USPSTF at the B grade or higher [33]. Thus, the USPSTF recommendations may impact access to services. In addition, evidence has shown that the current USPSTF guidelines have led to a decrease in clinicians recommending mammography to younger Black women [34]. Addressing this specific question, a recent simulation modeling study evaluated how the USPSTF screening mammography guidelines could be made more equitable for Black women in the US [35]. Simulation modeling was required because Black women have been historically underrepresented in screening trials, precluding analyses stratified by race [36]. Absent more representative trials that evaluate different starting ages for mammography, simulation studies and other post hoc approaches to estimating the comparative effectiveness of different screening strategies [32] provides the best possible evidence for guiding policy decisions. The authors reported that initiating biennial screening for Black women beginning at age 40 would achieve the same benefits of biennial screening beginning at age 50 observed for White women, which could reduce the Black-White difference in breast cancer mortality by 57% [35]. It should be noted that race-based approaches to medicine have been critiqued on multiple ethical and pragmatic grounds [37–39]. Nevertheless, if the USPSTF was to utilize a race-based approach when updating their screening guidelines to address the breast cancer disparity observed for younger Black women, instead of adopting more inclusive guidelines recommended by the ACR/NCCN/ASBrS, this could have implications for the allocation of mammography resources.

In addition to allocating mammography facilities on the basis of screening guidelines, more recent evidence has pointed to the importance of mammography facility quality [40, 41]. Quality measures for mammography facilities include academic setting, mammograms being read exclusively by breast imaging specialists, and the availability of digital mammography [40]. Designation as a Breast Imaging Center of Excellence (BICOE)^1^ by the ACR [42] has been used in prior research to understand the link between comprehensive assessments of breast imaging facilities and racial disparities in stage at diagnosis [41]. Importantly, community-based programs designed to equitably improve access to high-quality screening mammography facilities have been shown to meaningfully reduce disparities in breast cancer mortality. [22, 43]

Methods in spatial epidemiology and health geography may help improve mammography access through catchment analysis and improved resource allocation. We have previously demonstrated use of location-allocation techniques for increasing accessibility of HIV screening [44], and others have used catchment analyses for identifying disparities in access to mammography [45], albeit not in Delaware nor with a specific goal of reallocating services. As such, this study had two objectives related to evaluating and improving equitable access to screening mammography facilities in Delaware. The first objective was to conduct a statewide catchment analysis of mammography facilities to identify potential disparities in geographic access to breast cancer screening. By catchment, we mean proximity to the nearest mammography facility—this serves as a proxy for accessibility measured through driving time and/or administrative boundaries. The catchment analysis evaluated whether mammography facilities, including BICOE-accredited facilities, were spatially patterned by sociodemographic characteristics. Finding evidence of disparities by race and rurality, the second objective was to conduct a location-allocation analysis to identify candidate locations for the establishment of new mammography facilities to optimize equitable access according to the existing ACR guidelines and to the USPSTF guidelines with or without race-based considerations. We focused our analyses on Delaware because it is broadly representative of the US in terms of race and urban–rural characteristics [46, 47] and has among the highest incidence rates in the US for breast cancer among younger Black women [48] and triple negative breast cancer [49], an aggressive subtype of breast cancer that is more likely to present at a younger age [50]. In addition, Delaware has the cancer care infrastructure necessary to implement population-level prevention programs and a track record of eliminating other cancer disparities with improved screening programs. [51]

## Methods

### Data sources

Census tract measures of population size; number of women aged 40–49, 50–74, and older than 74; percentage of women who are Black; area deprivation; and percentage of households with at least one vehicle were obtained from the US Census Bureau’s American Community Survey 5-year estimates [52]. Area deprivation was operationalized as a Z-score composite of education, employment, income and poverty, and household composition, where a higher score indicates greater deprivation [53]. The per tract number of bus stops were obtained from the Delaware open data portal [54]. Mammography facilities were compiled from two sources, the U.S. Food and Drug Administration certified facility list [55] and the American College of Radiology’s accredited facility list [56], the latter resource also identifying whether a facility was a BICOE. We retrieved all sites in the Delaware state catchment area and, recognizing that patients may cross state lines for mammography services, we included sites within border-adjacent ZIP codes in Maryland and Pennsylvania to better estimate access for Delaware residents. For sites in Delaware, the number of active mammography units per site was obtained from the Delaware Department of Health and Social Services. This information was used to estimate site capacity, or how many screening mammograms a site could perform each year. Using a calculation by Young and colleagues [57], and the definition of maximum capacity of three mammograms per mammography unit per business hour [58], capacity of a facility with one mammography unit was estimated at an average of 4,500 screenings per unit per year. For sites with more than one mammography unit this value was multiplied by the number of units at that site. Lastly, census tract and county boundary definition shapefiles were downloaded from the U.S. Census Bureau [59]. Across Delaware’s three counties, we explored heterogeneity by tracts inside or outside of New Castle County (i.e., Kent and Sussex counties), as New Castle County is more urban and contains the relatively densely populated city of Wilmington, while Kent and Sussex counties tend to be more rural (see Additional file 1: Fig. S1).

### Statistical analysis

First, we used descriptive statistics to summarize statewide and county-specific census tract measures of population, transportation (a proxy for accessibility), and mammography sites. We then performed a catchment analysis using the ArcGIS Pro Service Area analytic tool to identify areas within 15, 30, and greater than 30 min driving time from each existing mammography site, with driving time serving as an indicator of geographic access. This Service Area analytic tool calculates the maximum driving distance from a point that can be traveled along a road network [60], representing a service catchment area.

As part of this catchment analysis, Poisson regression models predicted the expected number of mammography facilities, units, BICOE facilities, and BICOE units statewide and separately for New Castle County and Kent and Sussex Counties based on local population characteristics (enumerated earlier) to identify potential ecological disparities. All independent variables were standardized for modeling. Given the relatively small geographic size and population of Delaware, census tracts were chosen as our ecological unit to maximize statistical power in the regression models and improve precision of the location-allocation analysis (see below). These ecological models included the log of the census tract population of women aged 40 or more years as an offset to account for population differences in women eligible for screening mammograms. The estimates may be interpreted as relative risks per standard deviation change with corresponding 95% confidence intervals (CIs).

Next, we used the ArcGIS Pro Location-Allocation analytic tool to identify candidate locations for the placement of additional mammography sites in Delaware. This Location-Allocation tool uses heuristic procedures to identify locations for services based on location-specific demand [44]. Within this analysis, the services are mammography screenings, and demand represents the people eligible to receive these services according to screening guidelines. Three competing specifications of demand were used in these analyses. The primary specification was based on the USPSTF recommendation of biennial mammography screenings for women aged 50–74 years. [27] The second specification was based on the previously described simulation study by Chapman and colleagues that recommends initiating biennial screening in Black women at age 40, in addition to the USPSTF’s recommendation of biennial screening for all women aged 50–74 years. [35] The third specification was based on the ACR recommendation of annual mammograms for women age 40 and older [24]. These three specifications of demand were calculated for each census tract and represented the number of women who would be eligible to receive screening mammography each year. Population-weighted centroids were calculated for each census tract and represented the location of the women residing in that census tract (i.e., location of demand). New mammography sites could be placed anywhere within the census tracts, with candidate locations created using the ArcGIS Pro Fishnet tool. To identify locations of new mammography sites that would fill in the gaps in demand that the current mammography sites are unable to reach, the location-allocation analysis took into consideration the locations and the capacities of the existing mammography sites. This was achieved using the Maximize Capacitated Coverage problem type, which selects candidate sites such that the maximum amount of demand is served without exceeding the capacity of the sites [61]. Maximize Capacitated Coverage is similar to the Minimize Weighted Impedance (P-Median) problem type, with the addition of a capacity constraint. Candidate sites were assumed to have a single mammography unit with the same capacity as existing facilities with one unit, as this was the median of units for all facilities across the state. For all demand specifications, the location-allocation analyses were run three times, allowing for the addition of one, three, or five new mammography sites. Driving time from demand points to mammography sites was used to determine appropriate location allocation, and a cut-off of 20-min driving time was specified as the maximum amount of time an individual would likely travel to a site. Finally, we used the Location-Allocation tool to identify existing sites that might benefit from a conversion to a BICOE. This analysis used the primary USPSTF demand specification and all other parameters used in the previously described location-allocation analyses, with one difference: Only BICOE sites and their capacities were used as the existing locations, while all non-BICOE sites were specified as candidate locations.

All analyses were conducted in R version 3.6.3 (R Foundation for Statistical Computing, Vienna, Austria) and ArcGIS Pro version 2.9.0 (ESRI, Carrboro, NC). Computational codes in R are available to download from: https://doi.org/10.5281/zenodo.7958311.

## Results

Across 214 populated census tracts (250,811 women aged 40 or more years) in Delaware, there were 30 mammography facilities containing 44 total units. Statewide, 9 sites were BICOE (30% of all facilities) containing 20 units (45% of total units). New Castle County contained 16 facilities (53% of all facilities) with 25 units (57% of total units); 7 were BICOE sites (78% of all BICOE facilities) with 16 BICOE mammogram units (80% of all BICOE units). Kent and Sussex Counties contained 14 facilities (47% of all facilities) with 19 units (43% of total units); 2 were BICOE sites (22% of all BICOE facilities) with 4 BICOE units (20% of all BICOE units). There were 6 mammography facilities outside of Delaware but in bordering ZIP codes: 4 in Pennsylvania and 2 in Maryland. One of the Maryland sites was a BICOE.

Table 1 presents census tract measures of population, transportation, and mammography sites in Delaware overall and by county. On average by census tract, New Castle County had fewer women aged 50–74 years (649 versus 824) and over 74 years (155 versus 209), a higher proportion of Black women (26% versus 16%), a lower proportion of households with a vehicle (91% versus 95%), and a greater number of bus stops (14 versus 7) compared to Kent and Sussex Counties.

**Table 1:** Census tract measures of population, transportation, and mammography sites in Delaware overall and by county

Measure	Mean (standard deviation)		
	Delaware	New Castle county	Kent & Sussex counties
N. tracts	214	129	85
Population of women ≥ 40	250,811	140,374	110,437
Deprivation^a^	0 (1)	0 (1)	0 (1)
N. women 40–49	277 (188)	284 (193)	266 (181)
N. women 50–74	719 (384)	649 (328)	824 (437)
N. women > 74	176 (108)	155 (102)	209 (109)
Percent Black women	22 (22)	26 (25)	16 (14)
Percent w/ vehicles	93 (9)	91 (11)	95 (4)
N. bus stops	11 (10)	14 (11)	7 (8)
N. mammography sites	0.1 (0.4)	0.1 (0.4)	0.2 (0.5)
N. mammography units	0.2 (0.8)	0.2 (0.9)	0.2 (0.7)
N. BICOE sites	< 0.1 (0.2)	0.1 (0.3)	< 0.1 (0.2)
N. BICOE units	0.1 (0.7)	0.1 (0.8)	0.1 (0.3)

*BICOE* breast imaging center of excellence

^a^Operationalized as a Z-score composite of census tract indicators for education, employment, income and poverty, and household composition

### Catchment analysis

**Fig. 1:**
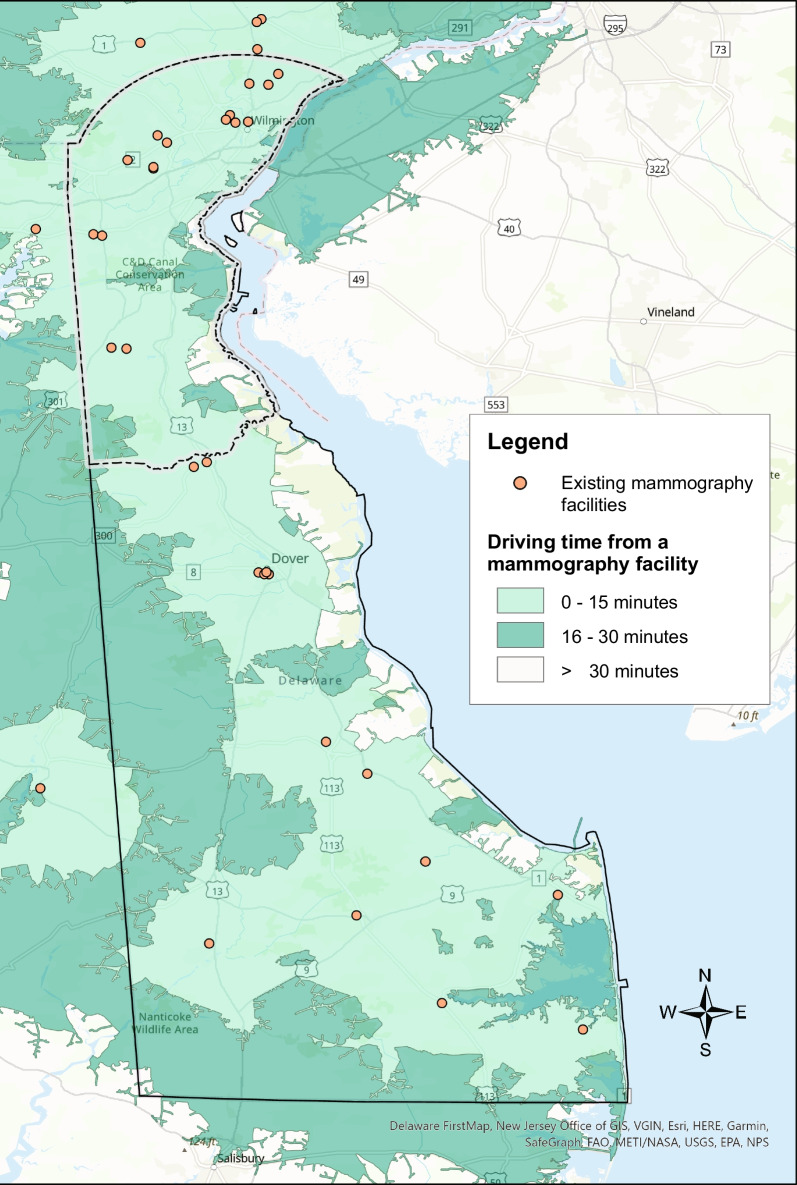
Delaware mammography facilities and average driving time from the population-weighted census tract centroid

The majority of Delaware’s population lives in the northernmost county, New Castle County, which encompasses 130 (61%) census tracts (Additional file 1: Fig. S1). Results of the service area analysis are illustrated in Fig. 1, showing a map of the existing mammography sites in Delaware, plus an additional six sites in adjacent ZIP codes, with shaded areas depicting the driving distance from each site. In New Castle County, 98% of census tracts are within 15 min, and none of the census tracts over 30 min driving time to a mammography site. Outside of New Castle County (Kent and Sussex) almost 78% of census tracts are within 15 min driving time, and just over 2% of census tracts are over 30 min driving time from a mammography site (Additional file 3: Table S1).

Table 2 and Additional file 3: Table S2 present the results of the census tract level Poisson regression models for the presence of mammography facilities and units and BICOE facilities and units, respectively, stratified by county. Several findings were apparent from these models. First, with every standard deviation increase in the number of women aged 40–49 in a given census tract, the number of mammography *facilities* increased 2.08 times (95% CI 1.25, 3.61) and 3.37 times (95% CI 1.48, 8.24) statewide and in New Castle County. With every standard deviation increase in the number of women aged 40–49 in a given census tract, the number of mammography *units* increased 2.30 times (95% CI 1.45, 3.78) and 5.63 times (95% CI 2.59, 13.3) statewide and in New Castle County. This trend was also observed for the number of BICOE facilities and units in Delaware and New Castle County. BICOE model results are not available for Kent and Sussex Counties due to the small number of BICOE facilities and units outside of New Castle County. Second, there was a trend toward fewer mammography facilities and units statewide and in New Castle County as the number of women aged 50–74 increased per census tract. Third, as the percentage of Black women in the census tracts increased, there were fewer mammography facilities and units statewide and by county. For example, for each standard deviation increase in Black women in a census tract, there were 68% fewer units in Delaware (95% CI 38%, 85%) and 89% fewer units in New Castle County (95% CI 62%, 97%). This finding was strongest for BICOE facilities and units. For example, for each standard deviation increase in Black women in a census tract, there were 89% fewer BICOE units in Delaware (95% CI 60%, 98%) and 99% fewer BICOE units in New Castle County (95% CI 86%, > 99%). Fourth, we also noted opposing associations for the transportation predictors, where a greater proportion of households with at least one vehicle was associated with a *decreased* rate of mammography facilities and units, and a greater number of bus stops was associated with an *increased* rate of mammography facilities and units.

**Table 2:** Poisson regression models predicting the number of mammography facilities and units by census tract measures in Delaware and by county. Estimates may be interpreted as relative risks with corresponding 95% confidence intervals. Bold font denotes statistical significance

Measure	Delaware		New Castle County		Kent & Sussex Counties	
	Number of facilities	Number of units	Number of facilities	Number of units	Number of facilities	Number of units
Deprivation^a^	1.16 (0.63, 2.07)	1.12 (0.66, 1.85)	1.34 (0.51, 3.10)	1.27 (0.56, 2.60)	0.64 (0.18, 2.29)	0.44 (0.14, 1.33)
N. women 40–49^b^	**2.08 (1.25, 3.61)**	**2.30 (1.45, 3.78)**	**3.37 (1.48, 8.24)**	**5.63 (2.59, 13.3)**	1.87 (0.91, 4.41)	**1.94 (1.01, 4.19)**
N. women 50–74^b^	0.72 (0.36, 1.36)	0.59 (0.32, 1.05)	0.41 (0.15, 1.06)	**0.25 (0.10, 0.58)**	0.88 (0.24, 2.67)	0.70 (0.22, 1.89)
N. women > 74^b^	1.01 (0.64, 1.53)	1.10 (0.75, 1.58)	1.23 (0.69, 2.09)	1.32 (0.81, 2.11)	0.93 (0.37, 2.01)	1.17 (0.57, 2.29)
Percent Black women^b^	**0.35 (0.14, 0.77)**	**0.32 (0.15, 0.62)**	**0.21 (0.05, 0.74)**	**0.11 (0.03, 0.38)**	0.54 (0.15, 1.50)	0.61 (0.22, 1.40)
Percent w/vehicles^b^	**0.42 (0.22, 0.82)**	**0.43 (0.25, 0.77)**	0.38 (0.12, 1.41)	0.35 (0.11, 1.36)	**0.47 (0.21, 0.98)**	**0.47 (0.24, 0.86)**
N. bus stops^b^	**1.63 (1.20, 2.17)**	**2.08 (1.67, 2.59)**	1.47 (0.94, 2.17)	**2.24 (1.67, 3.04)**	**1.96 (1.04, 3.86)**	**2.18 (1.28, 3.93)**

^a^Operationalized as a Z-score composite of census tract indicators for education, employment, income and poverty, and household composition

^b^Centered and scaled for modeling

### Location-allocation analysis

**Fig. 2:**
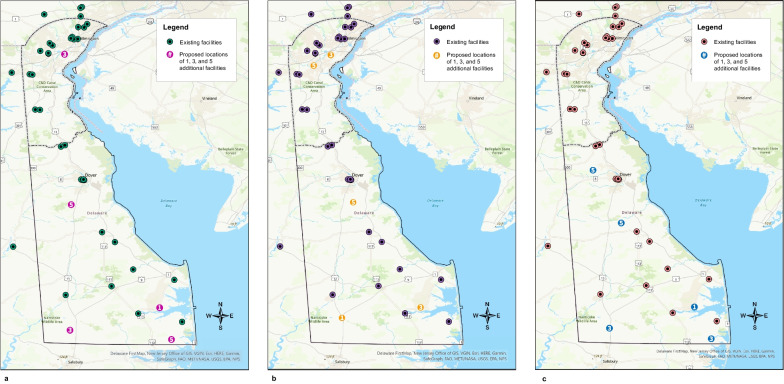
Results of the location-allocation analysis using the demand specifications for **a** all women per the U.S. Preventive Services Task Force mammography screening guideline, **b** all women per the U.S. Preventive Services Task Force mammography screening guideline plus biennial screening in Black women starting at age 40, and **c** all women per the American College of Radiology mammography screening guideline. Existing sites in Delaware and ZIP code adjacent locations in Pennsylvania and Maryland are shown as black/green circles. The numbered circles indicate where 1, 3, and 5 additional sites should be placed based on demand. These numbers are cumulative

Results of the location-allocation analysis using the USPSTF demand specification, the race-based specification, and the ACR specification are depicted in Fig. 2. Using the USPSTF demand specification, if adding one additional mammography site to the 36 existing sites within Delaware and the adjacent ZIP codes in Pennsylvania and Maryland, the best location based on demand would be in the southeast region of the state near the town of Millsboro. If adding three new sites, which would include the Millsboro location, one additional site would be placed in the southwestern region of the state near the town of Laurel, as well as in the northern region of the state, near the city of New Castle. And finally, when adding five new sites, which would include the three just described, one additional site would be placed in the southeastern region of the state near the town of Selbyville, and one in the center of the state near the town of Felton. Changing the demand specification to the race-based recommendations shifted the locations of the proposed mammography sites. Instead of three proposed sites in the southern region of the state, one central site, and one northern site, this analysis proposed two in the south near the towns of Laurel and Millsboro, one central near the town of Felton, and two in the north near the city of New Castle and the Bear area. Lastly, using the ACR specification, all five proposed sites fell outside of New Castle County: one west of Dover, one in the town of Milford, one in the town of Laurel, one near the town of Selbyville, and one near the town of Millsboro. Overall, the addition of five new mammography sites reduced driving time on average by 4% for the USPSTF and the race-based specifications, and by 2% for the ACR specification. New Castle County experienced the greatest improvement in driving time using the USPSTF and race-based specifications, with the addition of five new sites reducing average driving time by 8%. Using the ACR specification, Sussex County experienced the greatest improvement in driving time with a reduction of 12%.

Results from the BICOE location-allocation analysis using the USPSTF demand specification are depicted in Additional file 2: Fig. S2. Of the 10 existing BICOE sites, one is in Maryland, two are in Kent County, and seven are in New Castle County. The five existing non-BICOE sites identified by the location-allocation analysis for consideration for conversion to BICOE include one site in New Castle County (in the city of Wilmington) and four sites in Kent and Sussex Counties (in the towns of Millsboro, Rehoboth Beach, Seaford, and Milford).

## Discussion

In a catchment analysis of mammography access in Delaware, the state among the highest for rates of breast cancer among younger Black women in the USA, we observed what initially appeared to be adequate access to screening facilities. In New Castle County, the most populous county in the state, 98% of the population lived within a 15-min drive of a mammography facility. In the other, more rural two counties in the state, 78% and 98% of the population lived within a 15-min and 30-min drive of a facility, respectively. Across all racial groups, we observed a positive relationship between the number of younger women (i.e., 40–49 years) and the number of mammography facilities/units and BICOEs statewide. We did not observe significant associations between the number of women currently eligible for screening mammography under the current USPSTF guidelines and measures of mammography access, with the exception of a significantly decreased number of units relative to the number of women 50–74 years in New Castle County census tracts.

When mammography access was considered through a health equity lens, we found preliminary evidence suggestive of disparities in the allocation of mammography facilities and units related to census tract level measures of race and rurality. When examined statewide, for every standard deviation increase in the number of Black women in a census tract, there were 68% fewer mammography units. In New Castle County, the county with the largest Black population in the state, we observed 89% fewer units for every standard deviation increase in the number of Black women in a census tract. This finding was even stronger for BICOE units: for every standard deviation increase in the number of Black women in a census tract, there were 89% and 99% fewer BICOE units statewide and in New Castle County, respectively (with similar results observed for facilities). Fewer mammography facilities and units in predominantly Black census tracts points to a potential disparity in geographic access to screening facilities. Geographically, Delaware is a small state and therefore mammography is generally available within a reasonable driving distance. However, availability of services does not equate to accessibility of services, for example, if women are not provided time off from their job to get a mammogram. [62, 63]

Regarding disparities by SES, we did not find a significant association between area deprivation and the number of mammography facilities or units in New Castle County, Kent, and Sussex Counties, or statewide. Regarding disparities by rurality, the number of statewide facilities and units were proportional to the population size for New Castle County and Kent and Sussex Counties. However, while 100% of the census tracts in New Castle County were within a 30-min drive of a mammography facility, two census tracts in the southern part of Delaware had drive-times greater than 30 min. In addition, the more rural counties in the state accounted for 41% of the population but only 22% of the BICOEs.

The results of the location-allocation analysis using the USPSTF demand specification highlighted the opportunity to increase access in the more rural, southern part of the state. When adding five additional mammography sites, four were proposed for the southern part of the state and one in New Castle County. When five existing non-BICOE mammography facilities were considered for conversion to BICOE sites, four were identified in the southern part of the state and one in Wilmington, the largest city in the state. This finding is consistent with other research, which has found that among the greatest disparities in the geographic access to mammography facilities exist in small towns and rural areas [12, 57, 64]. When the results of these analyses are considered for the USPSTF guidelines with race-based screening demand specifications, three additional sites were proposed for the southern part of the state and two additional sites were proposed for New Castle County in areas that have larger numbers of younger Black and other minority women. Finally, under the ACR demand specification, all five new mammography sites were proposed for the southern, more rural part of the state.

This study, to our knowledge, represents the first location-allocation analysis of geographic access to screening mammography under multiple screening guideline demand specifications. Our results illustrate that decisions about allocating mammography screening resources are impacted by which set of screening guidelines are adopted. In this study, adopting ACR guidelines, which recommend all women initiate annual screening mammography beginning at age 40, would lead to a greater focus on improving access in rural areas. The USPSTF guidelines would lead to a similar allocation, albeit with a small shift in allocation to more populous areas. Adopting the USPSTF guidelines inclusive of a race-based approach to screening would lead to a greater allocation of mammography resources to more populous and racially diverse geographic areas. Replicating this type of analysis across a range of geographic regions that vary by land use, demographics, and population density would help to clarify whether this pattern of findings is generalizable or more idiosyncratic to a given place. That is, ACR guidelines may generally bias the allocation of resources to more rural areas and a race-based approach may generally bias the allocation of resources to areas with more Black women. In Delaware, these represent two different regions of the state. Conversely, there may be a greater convergence across guidelines in areas that, for example, are both rural and have larger relative Black populations (e.g., sections of the Mississippi Delta). [65]

This study has several limitations. First, our analyses focused only on Delaware and findings may not apply to other states or geographic regions, although the methods are transferrable [44]. In addition, we did not have access to data on the number of units per mammography facility for facilities outside of Delaware; this was assumed to be one unit based on the median number of units in Delaware. Delaware has notably elevated rates of breast cancer among Black women under age 50 [48], including rates of more aggressive subtypes of breast cancer [49], and therefore represents an important state in its own right to assess. However, this study did not consider other factors beyond spatial access to mammography screening that have been linked to racial disparities in breast cancer, such as exposures to breast cancer risk factors, tumor biology, and access to high-quality cancer care [3]. Beyond racial disparities, this study did not examine mammography access for other high-risk groups (e.g., Ashkenazi Jewish women). [66]

Second, drive time represented our proxy for accessibility, albeit without accounting for traffic. For women accessing mammography facilities via other means (e.g., public transportation) and for whom other barriers limit access (e.g., hours of operation, insurance, childcare) [67], our analysis may not fully capture these complex patterns. For example, while ownership of a vehicle was more limited in the urban areas of New Castle County, the number of bus stops was greater; one federally qualified health center in Wilmington previously noted that over 50% of their patients rely on busses for transit [68]. Therefore, future studies of access should consider the time it would take to reach a mammography site via public transportation and/or incorporating traffic time, as well as measures of other types of barriers, and mammography facility capacity. This research could inform the development of other interventions designed to close disparities in access to screening mammography, such as community outreach and transportation. The use of mobile mammography vans represents one such intervention that offers the potential to improve access to breast cancer screening for underserved communities [69]. However, additional research is needed to fully evaluate this approach [70] given patients ‘ perceived concerns about the quality of screening and other logistical challenges inherent to offering mobile services (e.g., follow-up care). [71]

A third limitation of this study was the use of BICOE designation as a quality measure. Prior research found that breast cancer diagnoses made at BICOE-designated facilities are less likely to be a later stage [41], but it remains unclear what explains this association. BICOE designation requires ACR accreditation in mammography and stereotactic biopsy, breast ultrasound and ultrasound-guided biopsy, and breast MRI and MRI biopsy or the ability to refer the patient for MRI/MRI biopsy to another facility with a referral relationship. Therefore, an ACR accredited mammography unit at a BICOE facility is not necessarily of higher quality than an ACR accredited unit at a non-BICOE facility. It may not be necessary, let alone feasible, to convert a mammography facility to a BICOE to improve access to mammography. There is also not an established relationship between BICOE-designated facility and radiologist characteristics. Separate research reported a relationship between radiologist characteristics (i.e., qualifications, affiliation, and experience) and false-negative rates (i.e., missed breast cancer detection), particularly for racial/ethnic minorities and lower-income women [72–74]. Last, there are multiple approaches for performing a catchment analysis, with no clear gold standard for all use cases. The choice of a specific method is often made based on investigator experience and preference, research needs, and computational requirements. Different approaches to catchment analysis may yield qualitatively different conclusions. [75, 76]

To conclude, drawing on the definition that health disparities represent potentially avoidable differences in disease outcomes [77], ensuring equitable geographic access to high-quality screening mammography facilities could help to close breast cancer disparities observed by race and rurality. However, making decisions about how to allocate mammography resources to ensure equitable access is contingent on which set of breast cancer screening guidelines are adopted, among other considerations (e.g., certificate of need). Given a set of guidelines, catchment and location-allocation analyses can guide the selection of locations for new mammography facilities and represent important methodological tools that can be leveraged in service of health equity. Future studies should collect additional data on access, quality, and capacity across geographic areas and population subgroups to facilitate the generation of more finely tuned and potentially impactful recommendations for the allocation of mammography facilities.

### Supplementary Information

**Additional file 1. **Heatmap of population density in Delaware.

**Additional file 2. **Results of the location-allocation analysis using the demand specification of all women per the U.S. Preventive Services Task Force mammography screening guideline, focusing only on Breast Imaging Centers of Excellence sites. Existing BICOE sites in Delaware and ZIP code adjacent locations in Pennsylvania and Maryland are shown as black dots. The numbered dots indicate where 1, 3, and 5 additional sites should be placed based on demand. These numbers are cumulative

**Additional file 3: Table S1.** Average driving time from the population-weighted census tract centroid to the nearest mammography facility in Delaware by county. **Table S2.** Poisson regression models predicting the number of Breast Imaging Centers of Excellence facilities and units by census tract measures in Delaware and by county. Estimates may be interpreted as relativerisks with corresponding 95% confidence intervals. Bold font denotes statistical significance.

## Data Availability

Computational codes in R are available to download from: https://doi.org/10.5281/zenodo.7958311. Datasets used for this study are referenced inline in the code and are based on publicly available sources; they may also be obtained from the corresponding author upon reasonable request.

## Footnotes

1 At the time of this publication, the ACR announced it will begin to transition the name for this designation from BICOE to ‘ACR Designated Comprehensive Breast Imaging Centers’.
