# Supplementary figures and images for: A catchment and location-allocation analysis of mammography access in Delaware, US: implications for disparities in geographic access to breast cancer screening
Source: Breast Cancer Res. 2023 Nov 8;25:137. doi: 10.1186/s13058-023-01738-w (PMC10631173; doi:10.1186/s13058-023-01738-w)

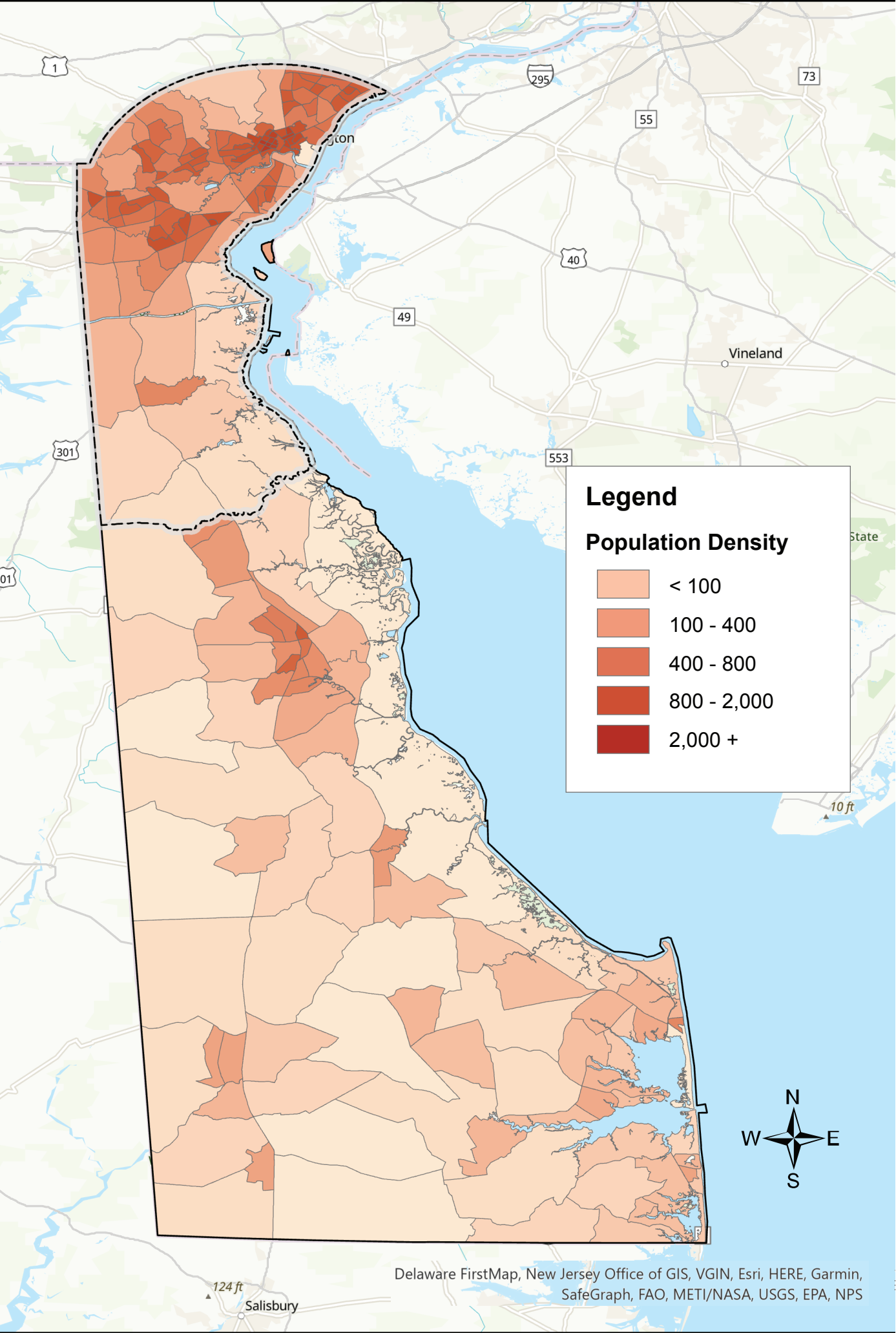

## Legend

### Population Density

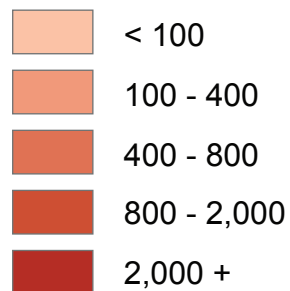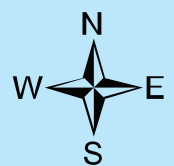

Supplement: Supplementary file 1 — Additional file 1. Heatmap of population density in Delaware. [file 13058_2023_1738_MOESM1_ESM.pdf]

**Location-allocation demand:  
US Preventive Services Task Force**

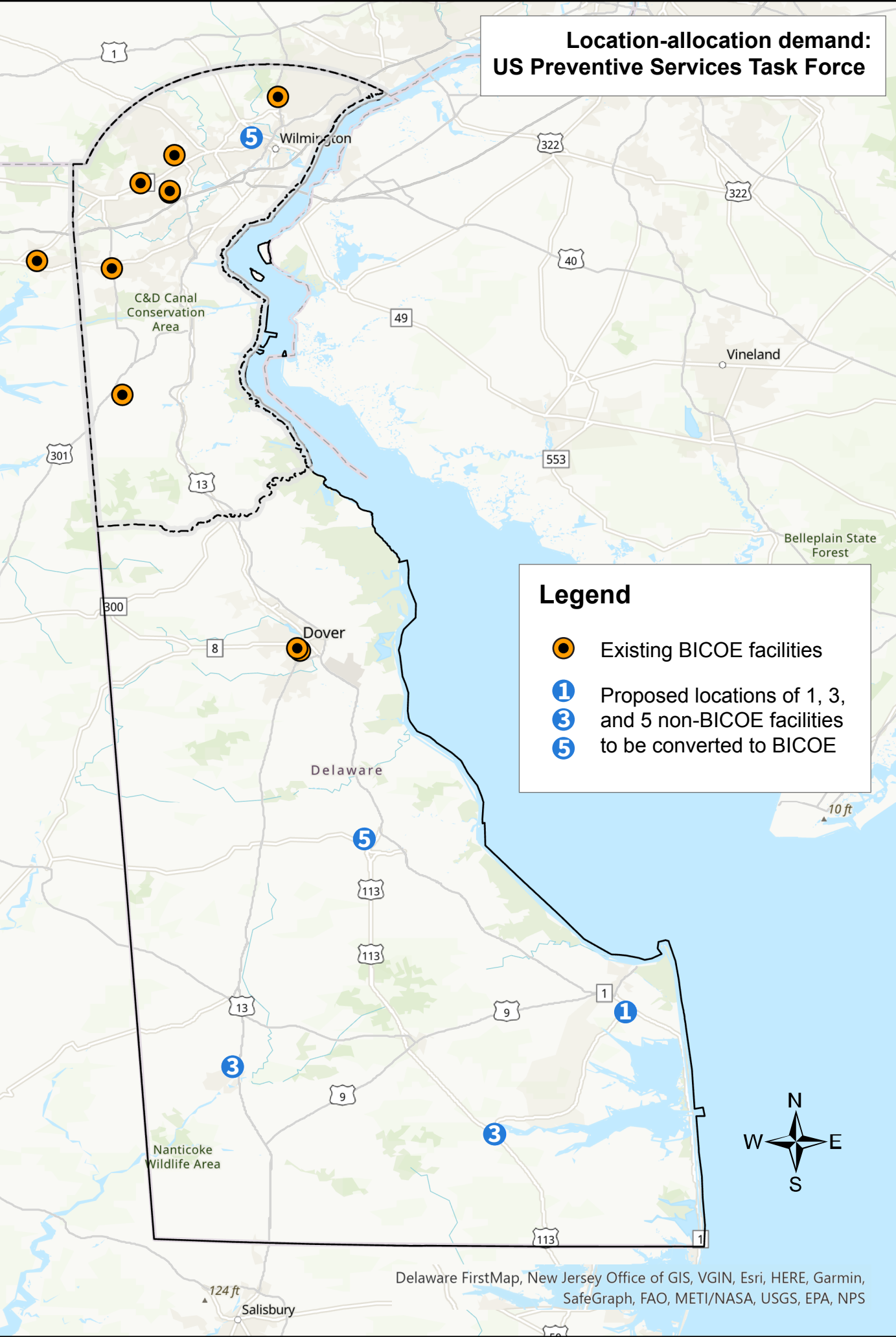

Supplement: Supplementary file 2 — Additional file 2. Results of the location-allocation analysis using the demand specification of all women per the U.S. Preventive Services Task Force mammography screening guideline, focusing only on Breast Imaging Centers of Excellence sites. Existing BICOE sites in Delaware and ZIP code adjacent locations in Pennsylvania and Maryland are shown as black dots. The numbered dots indicate where 1, 3, and 5 additional sites should be placed based on demand. These numbers are cumulative [file 13058_2023_1738_MOESM2_ESM.pdf]
