# Supplementary material for: A catchment and location-allocation analysis of mammography access in Delaware, US: implications for disparities in geographic access to breast cancer screening
Source: Breast Cancer Res. 2023 Nov 8;25:137. doi: 10.1186/s13058-023-01738-w (PMC10631173; doi:10.1186/s13058-023-01738-w)
Supplement: Supplementary file 3 — Additional file 3: Table S1. Average driving time from the population-weighted census tract centroid to the nearest mammography facility in Delaware by county. Table S2. Poisson regression models predicting the number of Breast Imaging Centers of Excellence facilities and units by census tract measures in Delaware and by county. Estimates may be interpreted as relativerisks with corresponding 95% confidence intervals. Bold font denotes statistical significance. [file 13058_2023_1738_MOESM3_ESM.docx]

**Supplemental Table 1**: Average driving time from the population-weighted census tract centroid to the nearest mammography facility in Delaware by county.

| **Service Area Catchment by County** | | | | |
| --- | --- | --- | --- | --- |
| Driving time to a mammography site | **New Castle** | | **Non-New Castle  (Kent and Sussex)** | |
|  | **n** | **%** | **n** | **%** |
| **0-15 minutes** | 127 | 97.7% | 66 | 77.7% |
| **15-30 minutes** | 3 | 2.3% | 17 | 20.0% |
| **30+ minutes** | 0 | 0% | 2 | 2.4% |

**Supplemental Table 2.** Poisson regression models predicting the number of Breast Imaging Centers of Excellence facilities and units by census tract measures in Delaware and by county. Estimates may be interpreted as relative risks with corresponding 95% confidence intervals. Bold font denotes statistical significance.

|  | **Delaware** | | **New Castle County** | | **Kent & Sussex Counties^a^** | |
| --- | --- | --- | --- | --- | --- | --- |
| **Measure** | Number of facilities | Number of units | Number of facilities | Number of units | Number of facilities | Number of units |
| Deprivation^b^ | 1.10 (0.30, 3.27) | 1.09 (0.41, 2.50) | 2.03 (0.37, 9.29) | 1.98 (0.47, 7.70) | -- | -- |
| N. women 40-49^c^ | **3.78 (1.27, 13.4)** | **6.72 (2.66, 19.2)** | **5.01 (1.27, 25.8)** | **16.6 (4.69, 74.4)** | -- | -- |
| N. women 50-74^c^ | 0.46 (0.09, 1.81) | **0.19 (0.05, 0.61)** | 0.50 (0.09, 2.45) | **0.14 (0.03, 0.48)** | -- | -- |
| N. women >74^c^ | 0.79 (0.31, 1.90) | 1.05 (0.55, 2.00) | 0.91 (0.34, 2.37) | 1.22 (0.54, 2.76) | -- | -- |
| Percent Black women^c^ | 0.16 (0.02, 0.78) | **0.11 (0.02, 0.40)** | **0.03 (0.00, 0.45)** | **0.01 (0.00, 0.14)** | -- | -- |
| Percent w/ vehicles^c^ | 0.31 (0.08, 1.41) | 0.31 (0.10, 1.02) | 1.16 (0.04, 115) | 1.41 (0.08, 59.2) | -- | -- |
| N. bus stops^c^ | **2.52 (1.57, 4.11)** | **3.26 (2.35, 4.69)** | **2.53 (1.29, 5.01)** | **4.47 (2.78, 7.99)** | -- | -- |

^a^ Models not fit due to the small number of Breast Imaging Centers of Excellence facilities and units outside of New Castle County.

^b^ Operationalized as a Z-score composite of census tract indicators for education, employment, income and poverty, and household composition.

^c^ Centered and scaled for modeling.
